# AMPI-AB validity and reliability: a multidimensional tool in resource-limited primary care settings

**DOI:** 10.1186/s12877-020-01508-9

**Published:** 2020-03-30

**Authors:** Marcos Daniel Saraiva, Amanda Lagreca Venys, Fábio Luiz Pantaleão Abdalla, Mariana Seabra Fernandes, Priscila Henriques Pisoli, Danilsa Margareth da Rocha Vilhena Sousa, Barbara Lobo Bianconi, Expedita Ângela Henrique, Vanessa Silva Suller Garcia, Lucas Henrique de Mendonça Maia, Gisele Sayuri Suzuki, Priscila Gonçalves Serrano, Marcel Hiratsuka, Claudia Szlejf, Wilson Jacob-Filho, Sérgio Márcio Pacheco Paschoal

**Affiliations:** 1 Medical Research Laboratory in Aging (LIM-66) of Geriatrics Division of the Clinics Hospital of São Paulo University (USP) Faculty of Medicine, São Paulo, Brazil; 2 Geraldo de Paula Souza Health Center School, USP Faculty of Public Health, São Paulo, Brazil; 3 Technical Area of Health of Older People, Municipal Health Secretariat of São Paulo, São Paulo, Brazil

**Keywords:** Comprehensive geriatric assessment, Screening tool, Primary care, Validation

## Abstract

**Background:**

The early identification of individuals at high risk for adverse outcomes by a Comprehensive Geriatric Assessment (CGA) in resource-limited primary care settings enables tailored treatments, however, the evidence concerning its benefits are still controversial. The main objective of this study was to examine the validity and reliability of the “Multidimensional Assessment of Older People in Primary Care (AMPI-AB)”, a CGA for primary care in resource-limited settings.

**Methods:**

Longitudinal study, with median follow-up time of 16 months. Older adults from a public primary care unit in São Paulo, Brazil, were consecutively admitted. Reliability was tested in a sample from a public geriatric outpatient clinic. Participants were classified by the AMPI-AB score as requiring a low, intermediate or high complexity of care. The Physical Frailty Phenotype was used to explore the AMPI-AB’s concurrent validity. Predictive validity was assessed with mortality, worsening of the functional status, hospitalizations, emergency room (ER) visits and falls. The area under the ROC curve and logistic regression were calculated for binary outcomes, and a Cox proportional hazards model was used for survival analysis.

**Results:**

Older adults (*n* = 317) with a median age of 80 (74–86) years, 67% female, were consecutively admitted. At the follow-up, 7.1% of participants had died, and increased dependency on basic and instrumental activities of daily living was detected in 8.9 and 41.1% of the participants, respectively. The AMPI-AB score was accurate in detecting frailty (area under the ROC curve = 0.851), predicted mortality (HR = 1.25, 95%CI = 1.13–1.39) and increased dependency on basic (OR = 1.26, 95%CI = 1.10–1.46) and instrumental (OR = 1.22, 95%CI = 1.12–1.34) activities of daily living, hospitalizations (OR = 2.05, 95%CI = 1.04–1.26), ER visits (OR = 1.20, 95%CI = 1.10–1.31) and falls (OR = 1.10, 95%CI = 1.01–1.20), all models adjusted for sex and years of schooling. Reliability was tested in a sample of 52 older adults with a median age of 72 (85–64) years, 63.5% female. The AMPI-AB also had good interrater (ICC = 0.87, 95%CI = 0.78–0.92), test-retest (ICC = 0.86, 95%CI = 0.76–0.93) and proxy reliability (ICC = 0.84, 95%CI = 0.67–0.93). The Cronbach’s alpha was 0.69, and the mean AMPI-AB administration time was 05:44 ± 02:42 min.

**Conclusion:**

The AMPI-AB is a valid and reliable tool for managing older adults in resource-limited primary care settings.

## Background

A comprehensive geriatric assessment (CGA) is a multidimensional diagnostic process focused on determining the social, physical, cognitive, psychological, and functional capabilities of older adults with the purpose of developing a coordinated and integrated management plan [1]. The early identification of individuals at high risk for adverse outcomes by a CGA enables tailored treatments and better allocation of resources [1, 2]. However, the evidence concerning the benefits of applying the broad principles of CGAs in primary care are controversial [3]. In a recent systematic review, CGAs based on primary care practice were acceptable to the individuals involved but had an inconsistent impact on mortality, functional status and hospital admission rates [4]. Moreover, there is a need for new models that address the complexity of care of older adults living with multimorbidity or frailty in primary care, as these models can lead to quality of care improvements without increases in costs [3–7].

In Brazil, primary care aims to provide universal access and comprehensive health care, to coordinate and to expand the coverage to more complex levels of care (e.g., specialized ambulatory care and in-hospital care), and to implement intersectoral actions for health promotion and disease prevention. Brazilian primary care clinics are focused on families and communities, integrating medical care with health promotion and public health actions [8]. To standardize the evaluation of older people according to the Brazilian primary care norms, specialists in Geriatrics, Gerontology and Public Health from the Secretariat of Municipal Health of São Paulo developed a CGA tool in 2015, named the Multidimensional Assessment of Older People (AMPI-AB). The main purpose of the AMPI-AB was to standardize the evaluation of older people at primary care units in the city of São Paulo. The detection of the main geriatric syndromes and individual risks among primary care older adults can guide healthcare plans targeting the maintenance of functional status, while considering the multidimensional complexity of the aging process.

The AMPI-AB is composed of 17 questions that can be applied by any trained healthcare professional. The questions are based on well-known and validated scores used to detect relevant geriatric problems, such as lack of social support, multimorbidity, polypharmacy, cognitive and sensory impairment, physical limitations, depression, falls, functional dependence, weight loss and poor oral health. The final score classifies older adults in low, intermediate or high complexity of care and guides individualized healthcare plans and referrals to secondary care. Despite the wide application of the AMPI-AB in São Paulo, no studies have validated the AMPI-AB. The objectives of this study were to examine the validity and reliability of the AMPI-AB in resource-limited primary care settings.

## Methods

### Study design and population

This longitudinal study included participants aged 60 years and older who were consecutively admitted to the public primary care unit Geraldo de Paula Souza of University of São Paulo Public Health School, in São Paulo, Brazil between August 2016 and February 2017. This unit is responsible for the primary care of approximately 4000 older adults from the west region of São Paulo. We excluded individuals with incomplete data. All subjects provided their written informed consent to their voluntary participation in this study and for the publication of their data. The study and informed consent form have been approved by the local ethics committee of São Paulo University Faculty of Medicine.

### Baseline measurements

At baseline, demographic characteristics were self-reported, and body mass index was calculated after anthropometry assessments at the primary care consultation. The AMPI-AB was applied by a multidisciplinary team composed of physicians, a nurse and a nutritional therapist. The instrument evaluates the following domains: age, self-rated health, social support, chronic conditions, polypharmacy, recent hospital admissions, falls, vision, hearing, physical limitations, cognition, humor, basic and instrumental activities of daily living, incontinence, unintentional weight loss and oral health. The total score ranges from 0 to 21, and initially proposed cutoffs, defined by specialists in Geriatrics, Gerontology and Public Health of the Secretariat of Municipal Health of São Paulo based on subject matter knowledge, classify the complexity of care as follows: low (0–5 points), intermediate (6–10 points) or high (≥ 11 points). The questions and the scoring method are detailed in Supplementary Figure 1.

Frailty defined according to the Physical Frailty Phenotype [9] was used to explore the concurrent validity of the AMPI-AB and was measured at baseline. We also assessed basic and instrumental activities of daily living (BADL and IADL) using the Katz and Lawton scales, respectively [10, 11]. Clinical conditions and drugs were obtained through chart reviews, allowing to calculate the Charlson Comorbidity Index to determine multimorbidity and to assess polypharmacy, respectively [12].

### Predictive validity: assessment of outcomes

Outcomes were assessed after 1 year by a structured telephone interview performed by two examiners blinded to the baseline assessments. If the participant had a diagnosis of dementia at baseline, the interview was conducted with a caregiver. Interviews assessed the occurrence of death, hospitalizations, emergency room (ER) visits and falls during the follow-up period. BADLs and IADLs were also evaluated using the Katz and Lawton scores, respectively. We considered mortality as the primary outcome and the other parameters as secondary outcomes.

### Time to complete the instrument and interrater, test-retest and proxy reliability

We tested the average time to complete the instrument and interrater, test-retest and proxy reliability in another consecutive sample of older adults (*n* = 52) cared in a public tertiary geriatric outpatient unit at the Clinics Hospital from University of São Paulo Medical School between December 2018 and April 2019. Two blinded examiners administered the AMPI-AB. To assess interrater reliability, the instruments were administered on the same day 4 h apart. To assess test-retest reliability, the instrument was administered twice by the same examiner within a 7-day interval. To assess proxy reliability, we applied the AMPI-AB by telephone to a proxy (who had contact with the participant at least once a week, including spouses, caregivers and family members) within 30 days after the participant’s in-person assessment. Time taken to complete the instrument was recorded in the first assessment for each participant and proxy.

### Analysis

In a post hoc study power calculation, we found that the study had 96% power to demonstrate a survival rate difference of 15% between older adults with low/intermediate complexity of care and older adults with high complexity of care, considering an allocation ratio of 5:1 between groups, 23 events, and an alpha-level of 0.05.

Characteristics of the participants according to the AMPI-AB classification in low/intermediate complexity of care versus high complexity of care were compared with the chi-square test and the Wilcoxon rank sum test for categorical and nonnormally distributed continuous variables. The accuracy of the AMPI-AB to discriminate frailty was investigated with a receiver operating characteristic (ROC) curve. The ROC curve was also used to discriminate the following dichotomous outcomes: acquired dependency in BADLs and IADLs (worsening disability), hospitalization, ER visits, and falls. Since the AMPI-AB’s original cut-off point determined by the specialists was arbitrary, the Youden index was calculated to determine the threshold that yields optimal discriminative performance. Additionally, we investigated the association of the AMPI-AB score with worsening disability, hospitalizations, ER visits and falls with logistic regression models adjusted for sex and years of schooling. We plotted Kaplan-Meier curves to illustrate the survival rate estimates for the AMPI-AB classification (low/intermediate complexity of care versus high complexity of care) and the log-rank test was used to compare the survival rate distributions between groups. We also investigated the association of the AMPI-AB total score and classification with mortality with Cox proportional hazards models, which were also adjusted for sex and years of schooling. The Schoenfeld residual test indicated that the proportional hazards assumption was met. To compare the predictive ability of the AMPI-AB with other well-established geriatric prognostic instruments, we also evaluated the association of the Charlson Comorbidity Index, Katz and Lawton scores with one-year mortality using Cox-proportional hazards models adjusted for age, sex and schooling years.

To assess interrater, test-retest and proxy reliability we calculated intraclass correlation coefficient (ICC) estimates and their 95% confident intervals (CI) for the AMPI-AB score based on a single-rating (two raters), absolute-agreement, 2-way random effects model [13]. The AMPI-AB categorical classification agreement was evaluated by the linearly weighted Kappa coefficient, and internal consistency was evaluated by Cronbach’s alpha [14]. All statistical tests were two-tailed, and an alpha level of 0.05 was used to determine significance. Analyses were performed using Stata version 15.1 (Stata Corp., College Station, TX).

## Results

We included 317 participants in the validity assessment, 67% female and 66% white, with a median follow-up time of 16 (16–14) months, median age of 80 (74–86) years, and median schooling duration of 8 (4–13) years. Sample baseline characteristics can be seen in Table 1. The median AMPI-AB score of the sample was 7 (9–4), and 16.4% of participants were classified as high complexity of care.

**Table 1:** Sample baseline characteristics according to AMPI-AB classification (low, intermediate or high complexity of care)

Baseline characteristics	Total (***n*** = 317)	Low ***n*** = 124 (39.12%)	Intermediate ***n*** = 141 (44.48%)	High ***n*** = 52 (16.40%)	***p***
**Age (years), median (IQR)**	80 (74–86)	75 (71–80.5)	83 (77–86)	85 (79.5–89.5)	< 0.001
**Females, n (%)**	211 (66.56)	79 (63.71)	98 (69.50)	34 (65.38)	0.596
**White individuals, n (%)**	209 (65.93)	80 (64.52)	96 (68.09)	33 (63.64)	0.786
**Schooling years, median (IQR)**	8.5 (4–13)	11 (4–15)	8 (4–13)	4 (3–11)	0.008
**BADL, median (IQR)**	12 (11–12)	12 (12–12)	12 (11–12)	10 (6–12)	< 0.001
**IADL, median (IQR)**	18 (15–18)	18 (18–18)	18 (14–18)	9 (1–17)	< 0.001
**Charlson comorbidity index, median (IQR)**	1 (0–2)	0 (0–1)	1 (0–2)	2 (1–3)	< 0.001
**Number of drugs consumed, median (IQR)**	5 (3–7)	3 (2–6)	5 (4–7)	7.5 (6–9)	< 0.001
**BMI, median (IQR)**	25.65 (22.72–28.34)	25.27 (22.86–27.91)	25.78 (22.5–28.81)	25.12 (22.04–28)	0.667

*AMPI-AB* Multidimensional Assessment of Older People in Primary Care, *IQR* Interquartile range, *BADL* Basic activities of daily living, *IADL* Instrumental activities of daily living, *BMI* Body mass index

### Concurrent validity

**Fig. 1:**
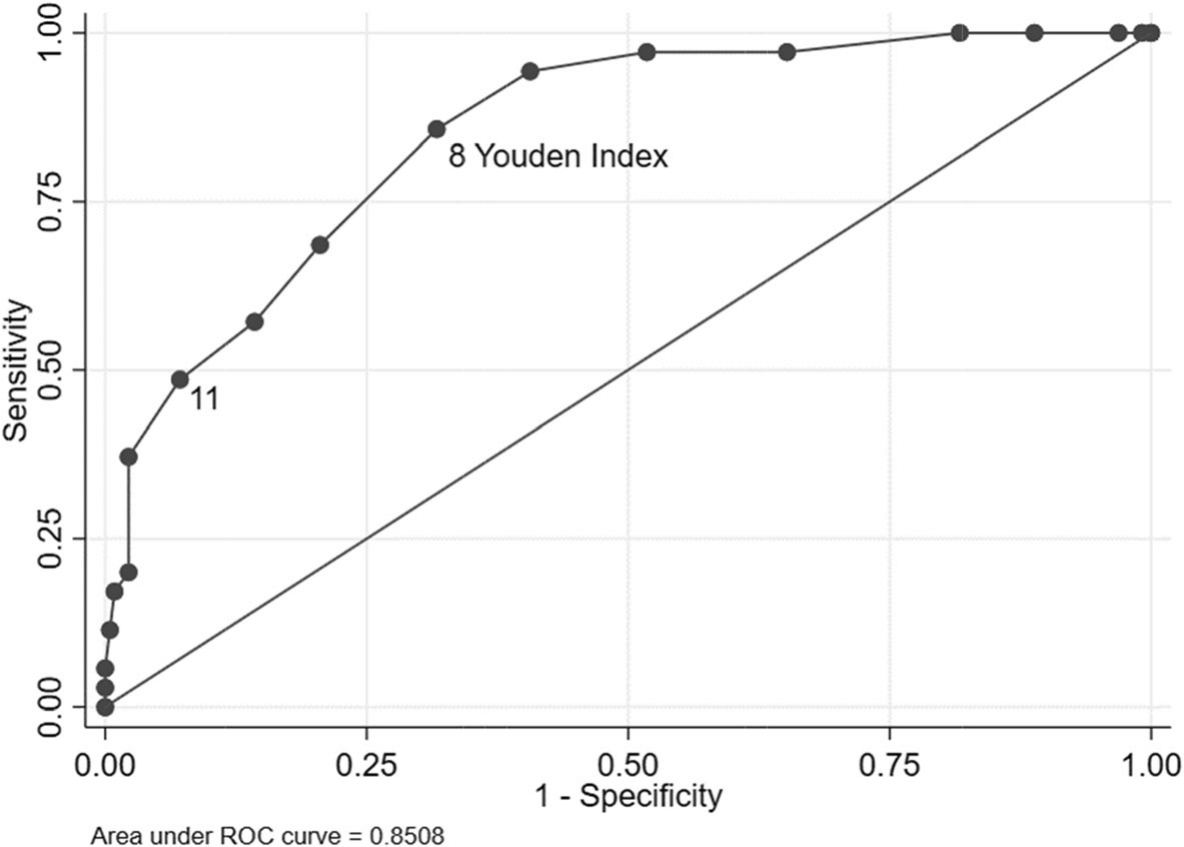
Area under the ROC curve for the Physical Frailty Phenotype diagnoses according to AMPI-AB score. The cutoff point of 11, initially proposed by consensus of specialists by the Secretariat of Municipal Health of São Paulo to classify a high complexity of care, had 48.6% sensitivity and 92.2% specificity. The cutoff point of 8, calculated by the Youden Index, had 85.7% sensitivity and 68.3% specificity

The median AMPI-AB score among frail (13.5%) and non-frail participants was 10 (12–8) and 6 (8–4), respectively (*p* < 0.001). The area under the ROC curve for the discrimination of frailty according to the AMPI-AB score was 0.851 (95%CI: 0.789–0.912). The cutoff point of 11, which was previously proposed by the Secretariat of Municipal Health, had 48.6% sensitivity and 92.2% specificity. The cutoff point of 8, calculated by the Youden Index, had 85.7% sensitivity and 68.3% specificity (Fig. 1). The AMPI-AB score was associated with frailty after adjustment for sex and schooling years (OR = 1.96, 95%CI = 1.43–2.68, *p* < 0.001).

### Predictive validity

**Fig. 2:**
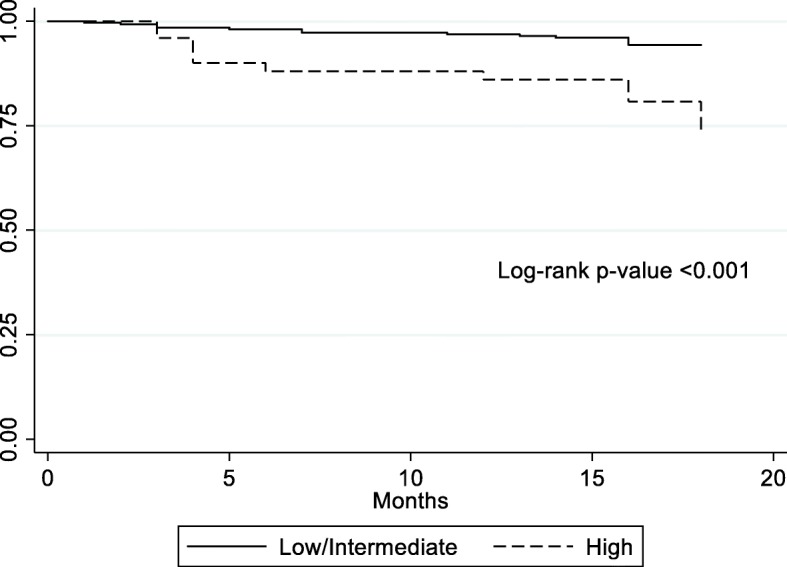
Kaplan-Meier survival rate curve according to the complexity of care (low/intermediate versus high complexity of care) as classified by the AMPI-AB. The log-rank test revealed a *p* value < 0.001

We could not contact 7 participants (2.2%) for the outcome evaluation. Baseline characteristics did not differ between the contacted and not contacted participants (results not shown). At follow-up, 7.1% of participants had died, and increased dependency in BADL and IADL was detected in 8.9 and 41.1% of the participants, respectively. Hospitalizations, ER visits and falls were reported in 18.5, 38.4 and 27.6% individuals, respectively. The survival rate in the high complexity of care participants was lower than in the low/intermediate complexity of care participants (log-rank test *p*-value < 0.001), as shown in Fig. 2. High complexity of care determined by the AMPI-AB (HR = 3.65, 95%CI = 1.56–8.54, *p* = 0.003) and the AMPI-AB total score (HR = 1.25, 95%CI = 1.13–1.39, *p* < 0.001) were both associated with mortality after adjustment.

The Charlson Comorbidity Index was also associated with mortality (HR = 1.28, 95%CI = 1.03–1.58, *p* = 0.027), however, BADL (HR = 1.01, 95%CI = 0.84–1.22, *p* = 0.869) and IADL (HR = 0.96, 95%CI = 0.88–1.04, *p* = 0.345) at baseline were not associated with mortality, after adjustment for age, sex and schooling years.

The association of the AMPI-AB total score with hospitalizations, worsening disability, ER visits and falls are shown in Table 2. Higher AMPI-AB scores were associated with all outcomes after adjustment.

**Table 2:** Association of the AMPI-AB score with the dichotomous outcomes and discriminative ability of the AMPI-AB

Outcome	Crude analysis OR (CI) p	Adjusted model^a^ OR (CI) p	Area under the ROC curve	Youden Index cutoff point
**BADL loss**	1.33 (1.18–1.51) *p* < 0.001	1.26 (1.10–1.46) p 0.001	0.77 ± 0.05	9
**IADL loss**	1.20 (1.11–1.30) *p* < 0.001	1.22 (1.12–1.34) *p* < 0.001	0.69 ± 0.03	7
**Hospitalizations**	1.15 (1.06–1.25) p 0.001	1.05 (1.04–1.26) p0.005	0.62 ± 0.04	7
**ER visits**	1.18 (1.09–1.27) *p* < 0.001	1.20 (1.10–1.31) *p* < 0.001	0.65 ± 0.03	7
**Falls**	1.09 (1.01–1.71) p 0.025	1.10 (1.01–1.20) p 0.027	0.59 ± 0.04	7

^a^Logistic regression models adjusted for sex and years of schooling

*BADL* Basic activities of daily living, *IADL* Instrumental activities of daily living, *ER* Emergency room

### Reliability and time to complete the instrument

To assess reliability, we included 52 participants, 63.5% female and 69% white, with a median age of 72 years (85–64) and a median schooling duration of 11 (4–15) years. The median AMPI-AB score was 5 (3–8). The mean time taken to complete the AMPI-AB was 05:44 ± 02:42 min. To assess proxy reliability, we contacted 24 proxies. The proxies took 06:16 ± 02:24 min on average to complete the AMPI-AB. Interrater reliability was assessed in 50 participants, with ICC = 0.87 (95%CI = 0.78–0.92). Test-retest reliability was assessed in 40 participants, with ICC = 0.86 (95%CI = 0.76–0.93). Finally, proxy reliability was assessed in 24 participants, with ICC = 0.84 (95%CI = 0.67–0.93). All of the ICCs showed good reliability [13]. Linearly weighted Kappa coefficients (95%CI) of the AMPI-AB categories for interrater, test-retest, and proxy reliability were 0.69 (0.64–0.84), 0.66 (0.41–0.91) and 0.76 (0.60–0.83), respectively. The Cronbach’s alpha was 0.69. All the aforementioned coefficients demonstrated substantial agreement [14].

## Discussion

In this study, the AMPI-AB was accurate in detecting physical frailty, and it predicted mortality, worsening disability, hospitalizations, ER visits and falls among older adults in primary care. Moreover, we demonstrated that the AMPI-AB had good reliability and agreement in interrater, test-retest and proxy reliability analyses.

To the extent of our knowledge, this is the first study to evaluate the AMPI-AB, an instrument for managing the healthcare of older people in a public health system in a lower-middle income country, developed by a consensus of specialists in public health, geriatrics and gerontology. The AMPI-AB aims (1) to identify older adults with high complexity of care needs who may benefit from specialized care and (2) to guide primary care professionals in the assessment of older adults and the formulation of individualized healthcare plans. Moreover, the AMPI-AB is a practical and simple instrument that does not require specialized equipment to assess the complexity of care, and it is a valuable option for managing older adults in resource-limited primary care settings in Latin America and worldwide.

The discriminative ability of several screening instruments to identify older persons at increased risk of functional decline and mortality ranges from 0.60 to 0.80 in the literature, which are very similar to those demonstrated by our study [4, 15–20]. However, none of these instruments have been evaluated in the context of resource-limited primary care settings. The Identification of Seniors At Risk – Primary Care (ISAR-PC) instrument is also a validated and easy-to-apply screening instrument to identify individuals at increased risk of functional decline in primary care. Through the assessment of age, dependence in IADL, and impaired memory, a study with 790 participants with median age of 75 years, showed that the ISAR-PC had an area under the ROC curve of 0.63–0.64 to discriminate functional decline in BADLs in 1 year. However, this study excluded patients with dementia, and 19% of participants were lost at follow-up [15].

According to international standards, Brazilian older adults have high access to primary care, however, many hospital admissions could be prevented by more effective prevention and treatment actions at this level of care [21]. Differences in the performance of primary care units are found and there is a continued need to update and adapt the model of healthcare delivery [21]. Low-income and lower-middle-income countries could benefit from the large-scale implementation of simple and short CGA instruments such as the AMPI-AB, that can be applied by any trained healthcare professionals, allowing the standardization of the assessment of older adults in primary care. Moreover, our study demonstrated that the AMPI-AB can also be applied by telephone with a proxy, facilitating the management of older adults with impaired access to health centers.

Nevertheless, our study results should be interpreted with caution due to some limitations. We used a convenience sample from a single primary care unit, and the sociodemographic profile of our population is not representative of the entire population of the city of São Paulo, hindering the generalizability of our results. Furthermore, outcomes such as functional status, falls and recent hospitalization are also components of the AMPI-AB, and this may have influenced the final result. However, the AMPI-AB assess functional status with less details than the instruments used to assess the outcomes. Moreover, we considered worsening disability as the outcome (the negative variation of functional status during follow-up) instead of the functional score per se. In addition, functional status at baseline alone was not a predictor of mortality in our study. Finally, the AMPI-AB is a multidimensional and complex instrument, and the main purpose of this paper was to validate the whole construct, and not individual items.

Future longitudinal studies with older adults from different Brazilian cities and other countries are required to confirm our data and extend the applicability of the AMPI-AB. Additionally, future studies should evaluate the ability of the AMPI-AB to manage the healthcare of older adults and its impact on a public health system, especially with regard to healthcare costs.

## Conclusions

In conclusion, the study presents evidence supporting the validity and reliability of the AMPI-AB for older adults in resource-limited primary care settings.

## Supplementary information

**Additional file 1: Figure S1.** Multidimensional Assessment of Older People in Primary Care (AMPI-AB).

## Data Availability

The datasets used and/or analyzed during the current study are available from the corresponding author on reasonable request.

## Abbreviations

AMPI-AB: Multidimensional Assessment of Older People in Primary Care
BADL: Basic activities of daily living
CGA: Comprehensive geriatric assessment
CI: Confident intervals
ER: Emergency room
HR: Hazard ratio
IADL: Instrumental activities of daily living
ICC: Intraclass correlation coefficient
IQR: Interquartile range.
ISAR-PC: Identification of Seniors At Risk – Primary Care
OR: Odds ratio
ROC: Receiver operating characteristic
