# Supplementary material for: AMPI-AB validity and reliability: a multidimensional tool in resource-limited primary care settings
Source: BMC Geriatr. 2020 Mar 30;20:124. doi: 10.1186/s12877-020-01508-9 (PMC7106646; doi:10.1186/s12877-020-01508-9)
Supplement: Supplementary file 1 — Additional file 1: Figure S1. Multidimensional Assessment of Older People in Primary Care (AMPI-AB). [file 12877_2020_1508_MOESM1_ESM.docx]

**SupPlementary Files**

Supplementary Figure 1. Multidimensional Assessment of Older People in Primary Care (AMPI-AB).

**
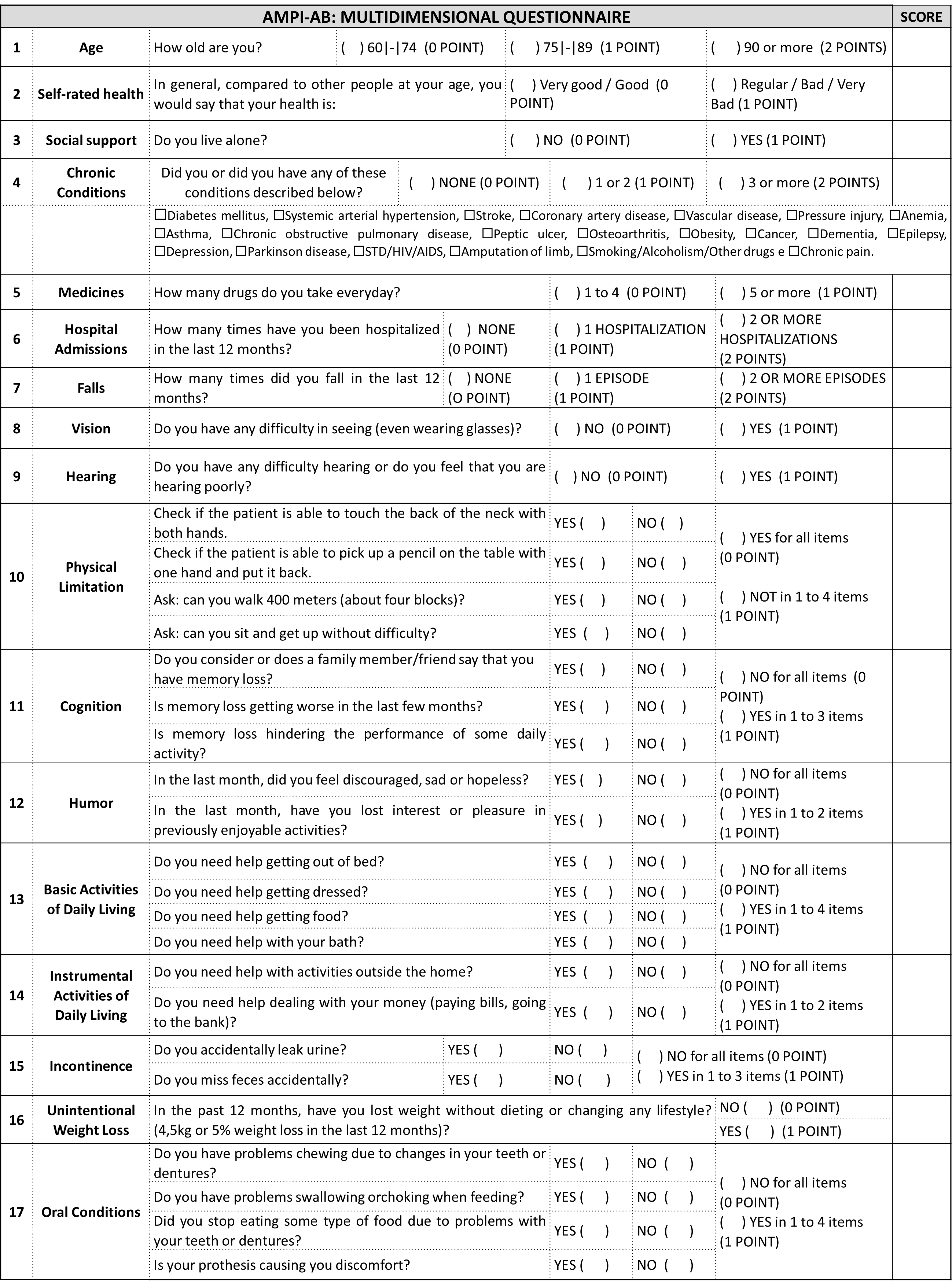
**
